# Recent Incarceration, Substance Use, Overdose, and Service Use Among People Who Use Drugs in Rural Communities

**DOI:** 10.1001/jamanetworkopen.2023.42222

**Published:** 2023-11-09

**Authors:** Daniel B. Hoover, P. Todd Korthuis, Elizabeth Needham Waddell, Canyon Foot, Caitlin Conway, Heidi M. Crane, Peter D. Friedmann, Vivian F. Go, Robin M. Nance, Mai T. Pho, Milan F. Satcher, Adams Sibley, Ryan P. Westergaard, April M. Young, Ryan Cook

**Affiliations:** 1 Department of Medicine, Section of Addiction Medicine, Oregon Health & Science University, Portland; 2 Oregon Health & Science University-Portland State University School of Public Health, Portland; 3 Division of General Internal Medicine, Oregon Health & Science University, Portland; 4 University of Wisconsin Madison, Madison; 5 Department of Medicine, University of Washington, Seattle; 6 Office of Research, University of Massachusetts Chan Medical School-Baystate, Baystate Health, Springfield; 7 Department of Health Behavior, University of North Carolina-Chapel Hill, Chapel Hill; 8 Department of Medicine, Section of Infectious Diseases and Global Health, University of Chicago, Chicago, Illinois; 9 Department of Community & Family Medicine, Dartmouth Health and Geisel School of Medicine at Dartmouth College, Hanover, New Hampshire; 10 Department of Medicine, University of Wisconsin-Madison, Madison; 11 College of Public Health, University of Kentucky, Lexington

## Abstract

**Importance:**

Drug use and incarceration have a substantial impact on rural communities, but factors associated with the incarceration of rural people who use drugs (PWUD) have not been thoroughly investigated.

**Objective:**

To characterize associations between recent incarceration, overdose, and substance use disorder (SUD) treatment access among rural PWUD.

**Design, Setting, and Participants:**

For this cross-sectional study, the Rural Opioid Initiative research consortium conducted a survey in geographically diverse rural counties with high rates of overdose across 10 US states (Illinois, Wisconsin, North Carolina, Oregon, Kentucky, West Virginia, Ohio, Massachusetts, New Hampshire, and Vermont) between January 25, 2018, and March 17, 2020, asking PWUD about their substance use, substance use treatment, and interactions with the criminal legal system. Participants were recruited through respondent-driven sampling in 8 rural US regions. Respondents who were willing to recruit additional respondents from their personal networks were enrolled at syringe service programs, community support organizations, and through direct community outreach; these so-called seed respondents then recruited others. Of 3044 respondents, 2935 included participants who resided in rural communities and reported past-30-day injection of any drug or use of opioids nonmedically via any route. Data were analyzed from February 8, 2022, to September 15, 2023.

**Exposure:**

Recent incarceration was the exposure of interest, defined as a report of incarceration in jail or prison for at least 1 day in the past 6 months.

**Main Outcomes and Measures:**

The associations between PWUD who were recently incarcerated and main outcomes of treatment use and overdose were examined using logistic regression.

**Results:**

Of 2935 participants, 1662 (56.6%) were male, 2496 (85.0%) were White; the mean (SD) age was 36 (10) years; and in the past 30 days, 2507 (85.4%) reported opioid use and 1663 (56.7%) reported injecting drugs daily. A total of 1224 participants (41.7%) reported recent incarceration, with a median (IQR) incarceration of 15 (3-60) days in the past 6 months. Recent incarceration was associated with past-6-month overdose (adjusted odds ratio [AOR], 1.38; 95% CI, 1.12-1.70) and recent SUD treatment (AOR, 1.62; 95% CI, 1.36-1.93) but not recent medication for opioid use disorder (MOUD; AOR, 1.03; 95% CI, 0.82-1.28) or currently carrying naloxone (AOR, 1.02; 95% CI, 0.86-1.21).

**Conclusions and Relevance:**

In this cross-sectional study of PWUD in rural areas, participants commonly experienced recent incarceration, which was not associated with MOUD, an effective and lifesaving treatment. The criminal legal system should implement effective SUD treatment in rural areas, including MOUD and provision of naloxone, to fully align with evidence-based SUD health care policies.

## Key Points

**Question:**

How do substance use treatment access, use of medication for opioid use disorder (MOUD), and overdose in rural areas differ for recently incarcerated people who use drugs (PWUD), compared with PWUD who have not been recently incarcerated?

**Findings:**

In this cross-sectional survey of 2935 PWUD in rural communities, 42% were recently incarcerated. Recent incarceration was associated with past-6-month overdose, substance use treatment, and not accessing treatment in the past 6 months but not treatment with MOUD or currently carrying naloxone.

**Meaning:**

The results of this study suggest that MOUD may be underused following incarceration in rural areas, despite clear evidence of benefit and support from multiple national organizations, and that the rural criminal legal system, especially jails and prisons, must urgently implement MOUD services.

## Teaser

This cross-sectional study analyzes data from the Rural Opioid Initiative research consortium survey to assess associations between recent incarceration, overdose, and substance use disorder treatment access among rural people who use drugs.

## Introduction

The US spends $80.7 billion annually on correctional agencies, including jails and prisons, excluding the associated cost burdens on incarcerated persons, their families, and their communities.^1,2^ The US drug prohibition policies have failed to discourage drug use or to curtail an increasingly dangerous contaminated drug supply and have contributed to mass incarceration that disproportionately harms Black or African American and Latinx populations.^3,4^ In the *Survey of Prison Inmates, 2016*,^5^ 47% of adults in custody met criteria for a substance use disorder (SUD) diagnosis. According to the 2019 census of jails, 15% of individuals in jail screened positive for opioid use disorder, but only 24% of jails allowed continuation of medications for opioid use disorder (MOUD) while in custody.^6^

In the 2 weeks following prison release, risk of opioid overdose is extremely high, and the risk of death from synthetic opioid overdose is 50 times greater than the general population.^7^ Medications for opioid use disorder, including US Food and Drug Administration–approved formulations of methadone, buprenorphine, and injectable naltrexone, are effective and lifesaving pharmacotherapy options and have been well-studied during incarceration.^8^ At a national level, the US has begun to recognize incarceration as an opportunity to begin SUD treatment and MOUD for eligible individuals, but improvement is slow, regionally variable, and faces particular barriers in rural areas.^9,10,11,12,13,14,15,16^

Despite rising rural pretrial incarceration, criminal legal involvement and incarceration of rural persons who use drugs (PWUD) have not been thoroughly described.^17^ A 2002 survey compared incarcerated PWUD from rural vs urban areas and found similar drug use, which was contrary to the authors’ hypothesis that rural environments would be protective against drug use.^18^ More recent publications were geographically small scale and limited to selected rural communities in Kentucky and Arkansas.^19,20,21^ The National Inmate Survey provides an epidemiological overview of incarcerated PWUD across the US but lacks detail about substance use behavior or any urban to rural comparison.^22^ Additionally, data collected during incarceration, as in the majority of the aforementioned sources, may be biased, since adults in custody fear to report drug use.^23^

Here, we present findings of a community-administered survey of PWUD in 65 rural counties across 8 rural regions in 10 states.^24^ This cross-sectional analysis investigated associations between exposure to recent incarceration and overdose, and engagement in SUD treatment, including MOUD.^24^ We hypothesized that markers of more severe SUD would be associated with higher rates of recent incarceration, and that, since incarceration is often disruptive to SUD care, recently incarcerated individuals would have more barriers to receipt of SUD treatment, as suggested by the Behavioral Model for Vulnerable Populations.^25^

## Methods

### Study Setting and Participants

The Rural Opioid Initiative (ROI) is a federally funded research consortium founded to assess drug use in 65 geographically diverse rural counties with high rates of overdose across 10 states (Illinois, Wisconsin, North Carolina, Oregon, Kentucky, West Virginia, Ohio, Massachusetts, New Hampshire, and Vermont).^26^ Each ROI site obtained approval from the local institutional review board for data collection and data sharing within the ROI consortium. Between January 25, 2018, and March 17, 2020, the ROI research sites conducted a baseline cross-sectional survey of PWUD in these communities. Trained study personnel obtained written informed consent. Participants were recruited through modified respondent-driven sampling (RDS) methods to capture this difficult to reach population.^27^ Recruitment first enrolled so-called seed respondents, who were willing to recruit additional participants from their networks. Seed respondents were identified at syringe service programs and community support organizations and through direct community outreach. These seed respondents and their recruits were given incentives ($40-$60) to participate and to recruit additional eligible participants ($10-$20 per participant), with a maximum of between 3 and 6 possible recruitments, depending on study site.^24^ Study participants were eligible if they had either injected any drug or used opioids nonmedically through any administration route in the past 30 days. Study participants were required to communicate in English and be at least 15 or 18 years of age, depending on study site. The reporting of the methods and results were guided by the Strengthening the Reporting of Observational Studies in Epidemiology (STROBE) reporting guideline checklist for cross-sectional studies.^28^

### Measures

Participants completed a baseline survey that included demographic characteristics, self-reported race, drug use behaviors, overdose, treatment history, involvement with the criminal legal system, housing status, and health insurance status. Race and ethnicity were included in the survey because race and ethnicity affect health behaviors and because of racially biased policies in the US War on Drugs. Participants chose 1 option of African; African American or Black; Alaskan Native; American Indian; Asian, Pacific Islander or Native Hawaiian; White; mixed race; or other (for analytic purposes, some of these groups were combined in our study). Participants who chose *other* were prompted to explain by free response. Participants were also asked if they identified as Hispanic or Latino. Participants were asked, “In the past 6 months how many days were you in jail or prison?” The primary exposure variable of interest, recent incarceration, was defined as having spent at least 1 day in prison or jail in the past 6 months. The survey also included questions regarding participants’ arrest history, number of times they were stopped by police, and community supervision.

Outcomes included SUD treatment experiences, history of naloxone ownership, and recent overdose. The SUD treatment experiences were assessed by asking participants if they had received “any treatment or help for an addiction problem” and if they had received buprenorphine sublingual formulations, extended-release buprenorphine, methadone, or extended-release naltrexone “for addiction treatment.” Both lifetime and past-30-day treatment experiences were assessed. Participants were also asked if they had any unsuccessful attempts to access any forms of SUD treatment, such as MOUD treatment, outpatient treatment, residential treatment, or withdrawal management. Lifetime history of naloxone receipt was measured with a single question. Participants also reported the approximate date of their most recent overdose, if ever, and those whose most recent overdoses were within 180 days of the survey date were classified as having an overdose in the past 6 months.

In addition to demographics, recent substance use, a binary indicator of any past-6-month homelessness, and having current health insurance were included as model covariates. To assess recent drug use, participants were asked which drugs they had ever used in their lifetime, and for any drug they reported having used, on how many of the past 30 days had they taken the drug “to get high.” Opioid use was defined as use of heroin, fentanyl, or opioid painkillers or nonmedical use of buprenorphine and methadone. Participants were also asked how often they injected any drugs in the past 30 days.

### Statistical Analysis

We first calculated descriptive statistics and bivariate associations (χ^2^ tests) between recent incarceration and respondent demographics and covariates. We also calculated RDS-weighted descriptive statistics of recent incarceration (by state), recent police stops, arrests, time in prison or jail, and probation or parole among participants.

Associations between recent incarceration and study dependent variables were estimated using multivariable mixed-effects logistic regression models. A model was fit for each of the following dependent variables: past-30-day SUD treatment experiences, past-30-day use of MOUD (among participants who reported past-30-day opioid use), failure to access treatment, lifetime naloxone access and current naloxone possession, and 6-month history of overdose. Models were adjusted for age, race, Hispanic ethnicity, gender (of survey options male/female/transgender/other), health insurance status, homelessness within the past 6 months, daily injection drug use, and past-30-day opioid, fentanyl, methamphetamine, and cocaine use. Adjustment variables were chosen a priori based on theorized associations with recent incarceration and the models’ dependent variables. The study site was included in all models as a random intercept. A 2-sided *P* < .05 was considered statistically significant. In order to correct for multiple hypothesis testing, all *P* values from outcome models were adjusted using the false discovery rate method; adjusted *P* values are reported as q values. All analyses were conducted from February 8, 2022, to September 15, 2023, using R, version 4.0.5 (R Project for Statistical Computing), with the lme4 and emmeans packages.

## Results

The ROI survey included a total of 3044 respondents. Of these respondents, 109 were missing information on recent incarceration, leaving 2935 participants. Four participants younger than 18 years were excluded, since juveniles interact with a different criminal-legal system than adults.

Respondents were mostly male (1662 [56.6%] vs 1257 female [42.8%] and 16 [0.6%] transgender, other, or declined) and White (2496 [85.0%] vs 89 Black or African American [3.0%], 209 American Indian [7.1%], and 141 mixed race, other, or declined to respond [4.8%]) with a mean (SD) age of 36 (10) years. In the previous 30 days, 2507 participants (85.4%) reported opioid use, 2178 participants (74.2%) used methamphetamine or amphetamine, 1105 participants (37.6%) used fentanyl, 1282 participants (43.7%) used cocaine, and 1663 participants (56.7%) injected drugs daily. This high proportion of injection drug use likely reflects sampling that included seed participants in syringe service programs. Compared with participants who were not recently incarcerated, recently incarcerated respondents were more likely to be male (61.4% vs 53.2%; *P* < .001), to be younger (≤45 years of age, 86.2% vs 77.1%; *P* < .001), and to have experienced homelessness within the last 6 months (62.7% vs 46.2%; *P* < .001) and were less likely to have health insurance (70.8% vs 76.9%; *P* < .001). Recently incarcerated participants also reported more methamphetamine use (82.3% vs 68.4%; *P* < .001) and more daily injection drug use (63.6% vs 51.7%; *P* < .001) (Table 1) than participants without recent incarceration. In total, 1039 recently incarcerated participants (84.9%) reported opioid use in the previous 30 days.

**Table 1.** Participant Characteristics and Substance Use Behaviors Overall and by Experience of Incarceration in the Past 6 Months

Characteristic	No. (%)			*P* value
	Overall (n = 2935)	Recently incarcerated (n = 1224)	Not recently incarcerated (n = 1711)	
Gender^a^				
Male	1662 (56.6)	752 (61.4)	910 (53.2)	<.001
Female	1257 (42.8)	464 (37.9)	793 (46.3)	
Transgender, other, or declined	16 (0.6)	8 (0.7)	8 (0.5)	
Age category, y				
<30	871 (29.7)	430 (35.1)	441 (25.8)	<.001
30-45	1503 (51.2)	625 (51.1)	878 (51.3)	
>45	561 (19.1)	169 (13.8)	392 (22.9)	
Race				
Black or African American	89 (3.0)	37 (3.0)	52 (3.0)	.02
American Indian	209 (7.1)	107 (8.7)	102 (6.0)	
White	2496 (85.0)	1014 (82.8)	1482 (86.6)	
Other, mixed race, or declined^b^	141 (4.8)	66 (5.4)	75 (4.4)	
Hispanic or Latinx	107 (3.6)	63 (5.1)	44 (2.6)	<.001
Experienced homelessness in past 6 mo	1558 (53.1)	768 (62.7)	790 (46.2)	<.001
Has health insurance	2183 (74.4)	867 (70.8)	1316 (76.9)	<.001
Substance use in past 30 d				
Opioids^c^	2507 (85.4)	1039 (84.9)	1468 (85.8)	.55
Fentanyl	1105 (37.6)	482 (39.4)	623 (36.4)	.11
Methamphetamine or amphetamine	2178 (74.2)	1007 (82.3)	1171 (68.4)	<.001
Cocaine	1282 (43.7)	535 (43.7)	747 (43.7)	.99
Daily injection	1663 (56.7)	778 (63.6)	885 (51.7)	<.001

^a^ For analytic models, gender was dichotomized as male vs nonmale due to small sample of participants categorized as transgender, other, or declined.

^b^ Mixed race was included as a survey option; other included response choices Asian, Pacific Islander, Native Hawaiian, or other; and 3 respondents declined to answer.

^c^ Opioid category included fentanyl.

Criminal legal involvement was common (Table 2). In the previous 6 months, an (RDS-weighted) estimated 42.2% of participants were incarcerated for at least 1 day in jail or prison (median [IQR], 13 [3-58] days), 23.4% had been arrested (median [IQR] 2, [1-5] arrests), 43.9% had been stopped by police (median [IQR], 3 [1-8] stops), and 28.3% were on probation or after prison supervision. There was substantial geographic variation: Wisconsin had the highest rate of recent incarceration (weighted estimate of 53.4%), while Ohio had the lowest (weighted estimate of 16.7%) (Figure).

**Table 2.** Unweighted and RDS Weighted Prevalence of Criminal Legal Involvement in the Past 6 Months

Characteristic	Unweighted No. (%)	RDS weighted %
Spent at least 1 d in prison or jail (No. of participants with non-missing data included, 2904)	1211 (41.7)	42.2
Duration of the recent incarceration, median (IQR), d	15 (3-60)	13 (3-58)
Recently arrested	832 (27.6)	23.4
No. of recent arrests, median (IQR)	2 (1-4)	2 (1-5)
Recently stopped by police	1472 (48.9)	43.9
No. of times recently stopped by police, median (IQR)	3 (2-6)	3 (1-8)
Recently on probation, parole, or supervision	969 (32.2)	28.3

**Abbreviation:** RDS, respondent-driven sampling.

**Figure.**
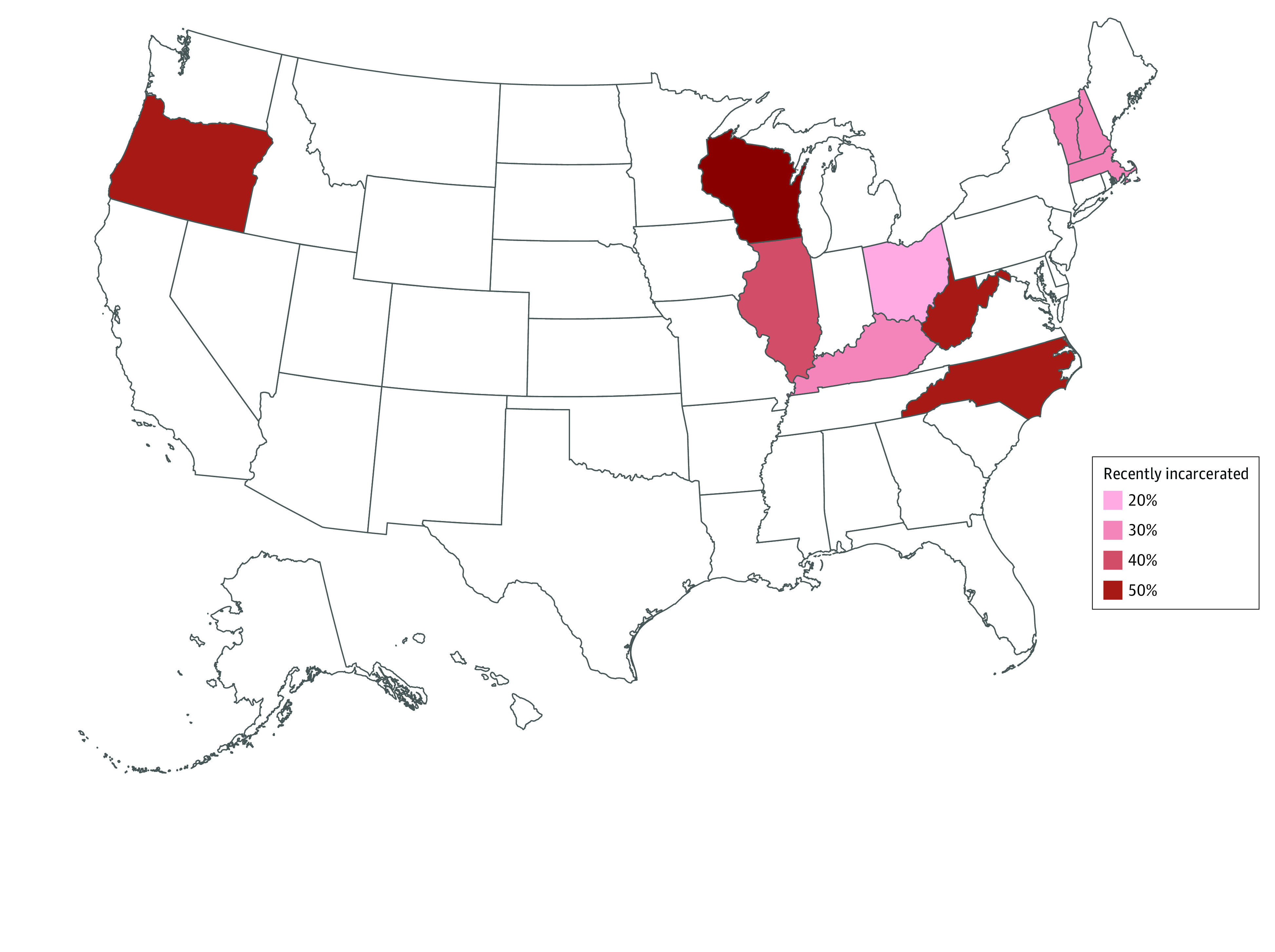
Weighted Prevalence of Recent Incarceration Among Participants in the Rural Opioid Initiative, by State Results are weighted to account for the respondent-driven sampling design.

Adjusted logistic regression models suggested that recent incarceration vs no recent incarceration was associated with higher past-30-day use of SUD treatment (39.1% vs 29.2%, adjusted odds ratio [AOR], 1.62 [95% CI, 1.36-1.93]; *P* < .001), but there was no evidence of higher engagement in past-30-day MOUD treatment (among 2507 participants who used opioids in the past 30 days; AOR, 1.03 [95% CI, 0.82-1.28; *P* = .81). Only 217 recently incarcerated participants who engaged in past 30-day opioid use (17.7%) reported past-30-day MOUD treatment. Recently incarcerated participants more commonly reported having tried and failed to access treatment in the past 6 months than participants without recent incarceration (47.1% vs 34.4%, AOR, 1.33 [95% CI, 1.13-1.57]; *P* < .001). Participants with recent incarceration also were more likely than participants without recent incarceration to report having ever received a naloxone kit (59.4% vs 50.2%; AOR, 1.28 [95% CI, 1.08-1.52]; *P* = .005) but not more likely to currently carry naloxone (38.0% vs 35.1%; AOR, 1.02 [95% CI, 0.86-1.21]; *P* = .83). Finally, recently incarcerated participants reported higher rates of overdose within the past 6 months than participants without recent incarceration (22.3% vs 15.3%; AOR, 1.38 [95% CI, 1.12-1.70]; *P* = .003). All associations continued to meet the threshold for statistical significance after adjustment for multiple testing (Table 3). Full model results are available in eTables 1, 2, 3, 4, 5, and 6 in Supplement 1.

**Table 3.** Adjusted Associations Between Recent Incarceration (Exposure Variable), Substance Use Treatment, and Overdose

Dependent variable	No. (%)		Adjusted odds ratio (95% CI)^a^	*P* value	q Value^b^
	Recently incarcerated (n = 1224)	Not recently incarcerated (n = 1711)			
Received SUD treatment in past 30 d^c^	471 (39.1)	494 (29.2)	1.62 (1.36-1.93)	<.001	<.001
MOUD treatment in past 30 d^d^	217 (17.7)	334 (19.4)	1.03 (0.82-1.28)	.81	.84
Tried and failed to access treatment in past 6 mo	576 (47.1)	589 (34.4)	1.33 (1.13-1.57)	<.001	.002
Ever received naloxone kit	727 (59.4)	859 (50.2)	1.28 (1.08-1.52)	.005	.008
Currently have naloxone	465 (38.0)	601 (35.1)	1.02 (0.86-1.21)	.83	.84
Overdose in past 6 mo^e^	266 (22.3)	255 (15.3)	1.38 (1.12-1.70)	.003	.005

Abbreviations: MOUD, medication for opioid use disorder; SUD, substance use disorder.

^a^ Adjusted for age, race, Hispanic ethnicity, gender, health insurance, past-6-month homelessness, injection frequency, and past-30-day opioid, fentanyl, methamphetamine, and cocaine use.

^b^ q Values are false discovery rate–adjusted *P* values.

^c^ A total of 2899 participants provided a response.

^d^ A total of 2507 participants reported past-30-day opioid use.

^e^ A total of 2864 participants provided a response.

## Discussion

This cross-sectional study comes at a time when many rural communities in the US are revising their approach to policies affecting PWUD, expanding community SUD treatment capacity, and advocating for evidence-based SUD treatment of adults in custody. Our findings highlight the severity of SUD among recently incarcerated PWUD in rural regions and missed opportunities for treatment engagement.

Recently incarcerated participants had a higher prevalence of injection drug use and overdose, suggestive of greater SUD severity. The high rate of recent overdose among recently incarcerated PWUD is both expected and alarming.^7,29^ A qualitative study of persons recently released from prison revealed an association between overdose risk and the distress generated by the challenges of reentry, such as surviving homelessness, managing exacerbated mental illness, and struggling to access health care.^29^ Disrupted social networks, stigma, and reduced opioid tolerance have also been highlighted as factors associated with overdose after release and may be magnified in rural communities.^30^ Recently incarcerated individuals included in the present analysis were unfortunately not any more likely to currently carry naloxone. A Justice Community Opioid Innovation Network survey of 185 jails in counties with high rates of opioid overdose found that only 30% of participating jails reported offering naloxone at release.^9^ Naloxone distribution should become standard practice at release.

Longer duration of substance use leading to the development of severe SUD could explain the increased SUD treatment use among individuals recently incarcerated. The survey did not ask whether SUD treatment reported was related to court mandates or to referrals at release, but recently incarcerated individuals did more commonly report trying and failing to access treatment. Past-30-day MOUD treatment was not associated with recent incarceration, despite the positive association for past-30-day SUD treatment. Historically, the criminal legal system has not offered opioid-agonist MOUD, so incarceration often disrupts recovery plans including MOUD. Forced discontinuation of MOUD during incarceration is extremely stressful, adds to other stresses during incarceration, and negatively impacts recovery from SUD and overdose risk after release.^31,32^ During the survey time frame, jails across the country rarely provided MOUD services; as of 2022, jail implementation of best practice recommendations for MOUD and SUD services remained variable.^10,14,16^ In the present study, recently incarcerated PWUD reported more recent overdoses but no increased engagement in MOUD treatment. Although 84.9% of recently incarcerated PWUD reported opioid use, only 17.7% received MOUD treatment in the past month. These gaps suggest missed opportunities to initiate MOUD prior to release from incarceration.

According to a 2021 O’Neill Institute report, many US states are taking actions promoting MOUD for adults in custody.^33^ Nine bills were introduced during the 2020 to 2021 period, and at least 28 states had guiding executive orders related to SUD treatment and incarceration. But the existing framework is inadequate and restrictive in some cases, such as allowing only injectable extended-release naltrexone rather than all US Food and Drug Administration–approved MOUD.^9,33^ A 2019 to 2021 survey of 185 jails in counties with high rates of opioid overdose found that MOUD was mostly provided to pregnant persons and individuals already taking MOUD when booked into jail; new inductions to MOUD were less available and were implemented close to release.^9^ Methadone is the least available MOUD for withdrawal management and new inductions in jail, despite having the strongest evidence for reducing recidivism and improving health care outcomes.^8,9,10,34^ Regulatory barriers deter jails from directly providing methadone.^35^ Methadone dispensaries can partner with local jails, but methadone dispensaries tend to be farther away and harder to access in rural communities.^36^ Urgent action is needed by the US Drug Enforcement Administration to develop a new pathway for methadone dispensation by jails.^35^

In 2019, New Jersey funded a statewide technical assistance effort that required jails to develop MOUD implementation plans. A 2020 survey of New Jersey jail MOUD practices suggests that this initiative was partially successful, but individual jails restricted the scope of MOUD to what they perceived as most effective or feasible for their environment—for example, only offering a buprenorphine taper without option to continue buprenorphine, and then directing all candidates to extended-release naltrexone for maintenance.^10^ For individuals with lower-level charges, jails have a diminishing window to provide relevant SUD and reentry services, which was an additional challenge observed in New Jersey’s implementation.^10^ Applying lessons learned in New Jersey, the path forward must begin with aligning goals for patient-centered MOUD treatment of individuals in the criminal legal system with best practices in addiction treatment, and follow with education, implementation assistance, accountability, and sustainable funding.

Lack of criminal legal system funding apportioned for SUD treatment and lack of health insurance coverage during incarceration further contribute to poor SUD treatment access for this population. The present study found that recently incarcerated individuals were more commonly uninsured. Notably, Medicaid must be reactivated after incarceration, and some jails have dedicated staff to coordinate this, but inconsistent implementation creates barriers to health care and SUD treatment on reentry.^16,37^ Inequitable access to care could be prevented by (1) providing prerelease access to Medicaid coverage via the Centers for Medicare and Medicaid Services approval of state-specific 1115 Demonstrations, (2) enacting the Medicaid Reentry Act of 2021, or (3) reversing the federal Medicaid exclusion of people who are incarcerated.^38,39,40^

We found substantial criminal legal system involvement beyond incarceration within this sample. Nearly a third (28.3%) of individuals recently incarcerated reported enrollment in community supervision programs, which could ideally function as a referral nexus for local health care, SUD treatment, and social services including housing.^41,42^ On any given day in the US, 374 000 persons are incarcerated for a drug offense, which accounts for 20% of the incarcerated population in the US, but SUD treatment coordination with probation and parole departments and expansion of incarceration diversion programs could reduce these figures.^43^ Seattle’s Law Enforcement Assisted Diversion is a prebooking program that diverts low-level behavioral and drug-related offenses to intensive case management and community services.^44^ This program reduces arrests, reduces exposure to incarceration, and saves about $2000 in legal costs per client.^44,45^

### Limitations

This study has limitations. First, the number of days incarcerated suggests more likelihood of recent jail exposure vs prison exposure, but the survey did not distinguish postrelease from jail vs postrelease from prison even though the 2 circumstances are quite different. Second, the study assessed behaviors and SUD services access in the past 6 months, generally, without further granularity of time frame. Due to the survey methods, it is unknown whether treatment experiences preceded or followed the recent incarceration episodes. Third, this analysis did not attempt to explain geographic variation in findings. Without a paired analysis of services in each state’s jails and prisons, we could not correlate participants’ responses to any specific SUD treatment services being provided (or not provided) by their local jails and prisons. Local regional diversion programming, jail-based MOUD services for adults in custody, state-level Medicaid administration, and re-entry programs are all associated with the experiences of PWUD with criminal legal system involvement. Fourth, overall and state-specific prevalence of criminal-legal involvement were derived using RDS weighting and should be interpreted with caution. Although RDS is a validated method of sampling hard to reach populations, such as that analyzed in the present study, there are assumptions underlying its accuracy that were unlikely to be met in the ROI.^24^

## Conclusions

This cross-sectional study of PWUD in rural communities found a high prevalence of incarceration and that recent incarceration was associated with both markers of more severe SUD and heightened barriers to accessing SUD treatment. Although there is a robust evidence base within health care, further implementation research is needed to develop the best care strategies for adults in custody with SUD and to overcome service access disparities within the criminal legal system that are amplified within rural areas. Urgent action must be taken to standardize and disseminate best practice implementation of MOUD and increase the rate of prerelease MOUD inductions and naloxone provision to prevent overdose deaths. Outside of incarceration, community health care professionals and community-based social service agencies should partner with community corrections departments to address structural factors and treat SUDs. Further advocacy is needed to realize the ethical necessity of SUD treatment, including MOUD for adults in custody, destigmatize SUD within the criminal legal system, convince state leaders of the cost-effectiveness of implementing MOUD, and couple state-level mandates with funding and technical assistance.

## Notes

Supplement 1. **eTable 1.** Model 1: Received SUD treatment in past 30 days (N = 2899) **eTable 2.** Model 2: Past 30-day receipt of MOUD (N = 2505 participants who reported past 30-day opioid use) **eTable 3.** Model 3: Tried and failed to access SUD treatment in past 6 months (N = 2935) **eTable 4.** Model 4: Ever received naloxone **eTable 5.** Model 5: Currently have naloxone **eTable 6.** Model 6: Overdose in past 6 months (N = 2867)

Supplement 2.
Data Sharing Statement
