# Error in Grant Number

**DOI:** 10.1001/jamanetworkopen.2023.48851

**Published:** 2023-12-07

In the Original Investigation titled “Recent Incarceration, Substance Use, Overdose, and Service Use Among People Who Use Drugs in Rural Communities,”^1^ published November 9, 2023, there was a typographic error in a grant number in the Funding/Support paragraph. The grant number now reads, “UG3DA044822/UH3DA044822.” This article has been corrected.^1^
