# Supplementary material for: Recent Incarceration, Substance Use, Overdose, and Service Use Among People Who Use Drugs in Rural Communities
Source: JAMA Netw Open. 2023 Nov 9;6(11):e2342222. doi: 10.1001/jamanetworkopen.2023.42222 (PMC10636631; doi:10.1001/jamanetworkopen.2023.42222)
Supplement: Supplement 1. — eTable 1. Model 1: Received SUD treatment in past 30 days (N = 2899) eTable 2. Model 2: Past 30-day receipt of MOUD (N = 2505 participants who reported past 30-day opioid use) eTable 3. Model 3: Tried and failed to access SUD treatment in past 6 months (N = 2935) eTable 4. Model 4: Ever received naloxone eTable 5. Model 5: Currently have naloxone eTable 6. Model 6: Overdose in past 6 months (N = 2867) [file jamanetwopen-e2342222-s001.pdf]

Supplementary Online Content

Hoover D, Korthuis PT, Waddell EN, et al. Recent incarceration, substance use, overdose, and service use among people who use drugs in rural communities. *JAMA Netw Open*. 2023;6(11):e2342222. doi:10.1001/jamanetworkopen.2023.42222

eTable 1. Model 1: Received SUD treatment in past 30 days (N = 2899)
eTable 2. Model 2: Past 30-day receipt of MOUD (N = 2505 participants who reported past 30-day opioid use)
eTable 3. Model 3: Tried and failed to access SUD treatment in past 6 months (N = 2935)
eTable 4. Model 4: Ever received naloxone
eTable 5. Model 5: Currently have naloxone
eTable 6. Model 6: Overdose in past 6 months (N = 2867)

This supplementary material has been provided by the authors to give readers additional information about their work.

© 2023 Hoover DB et al. *JAMA Network Open*.

eTable 1. Model 1: Received SUD treatment in past 30 days (N = 2899)

term	estimate	std.error	statistic	p.value
(Intercept)	-1.755			
Recent Incarceration	0.481	0.09	5.344	<.001
Health insurance	0.985	0.116	8.503	<.001
Age				
18-30	Ref			
31 - 45	-0.026	0.098	-0.264	0.792
46+	-0.563	0.134	-4.2	<.001
Race				
Black	Ref			
Native American	-0.314	0.312	-1.007	0.314
Other/Mixed race/Refused	0.167	0.324	0.515	0.607
White	0.135	0.255	0.53	0.596
Male	-0.018	0.087	-0.203	0.839
Hispanic	-0.184	0.247	-0.744	0.457
Homeless in past 6 months	-0.067	0.088	-0.759	0.448
Injected drugs daily in past 30 days	-0.364	0.09	-4.039	<.001
Past 30-day drug use				
Methamphetamine	-0.02	0.114	-0.177	0.86
Cocaine	-0.011	0.097	-0.11	0.913
Fentanyl	-0.049	0.103	-0.473	0.636
Any opioids	0.249	0.134	1.865	0.062
SD (site-level intercept)	0.623			

© 2023 Hoover DB et al. *JAMA Network Open*.

eTable 2. Model 2: Past 30-day receipt of MOUD (N = 2505 participants who reported past 30-day opioid use)

term	estimate	std.error	statistic	p.value
(Intercept)	-2.75			
Recent Incarceration	0.027	0.114	0.236	0.813
Health insurance	1.258	0.172	7.3	<.001
Age				
18-30				
31 - 45	-0.023	0.122	-0.186	0.852
46+	-0.508	0.169	-3.003	0.003
Race				
Black				
Native American	0.024	0.44	0.054	0.957
Other/Mixed race/Refused	0.362	0.438	0.827	0.408
White	0.562	0.36	1.562	0.118
Male	-0.125	0.107	-1.168	0.243
Hispanic	0.224	0.286	0.783	0.434
Homeless in past 6 months	-0.1	0.109	-0.91	0.363
Injected drugs daily in past 30 days	-0.183	0.112	-1.63	0.103
Past 30-day drug use				
Methamphetamine	-0.229	0.133	-1.723	0.085
Cocaine	0.183	0.12	1.534	0.125
Fentanyl	0.021	0.121	0.175	0.861
SD (site-level intercept)	0.558			

© 2023 Hoover DB et al. *JAMA Network Open*.

eTable 3. Model 3: Tried and failed to access SUD treatment in past 6 months (N = 2935)

term	estimate	std.error	statistic	p.value
(Intercept)	-1.552			
Recent Incarceration	0.289	0.085	3.393	0.001
Health insurance	0.111	0.1	1.116	0.264
Age				
18-30				
31 - 45	-0.172	0.093	-1.844	0.065
46+	-0.491	0.127	-3.876	<.001
Race				
Black				
Native American	-0.281	0.285	-0.985	0.324
Other/Mixed race/Refused	-0.106	0.304	-0.348	0.728
White	-0.222	0.238	-0.933	0.351
Male	-0.07	0.083	-0.845	0.398
Hispanic	0.209	0.227	0.92	0.357
Homeless in past 6 months	0.254	0.084	3.027	0.002
Injected drugs daily in past 30 days	0.283	0.086	3.279	0.001
Past 30-day drug use				
Methamphetamine	0.073	0.11	0.668	0.504
Cocaine	0.156	0.092	1.701	0.089
Fentanyl	0.354	0.098	3.603	<.001
Any opioids	0.619	0.132	4.695	<.001
SD (site-level intercept)	0.506			

© 2023 Hoover DB et al. *JAMA Network Open*.

eTable 4. Model 4: Ever received naloxone

term	estimate	std.error	statistic	p.value
(Intercept)	-1.541			
Recent Incarceration	0.244	0.087	2.798	0.005
Health insurance	0.235	0.101	2.334	0.02
Age				
18-30				
31 - 45	-0.055	0.096	-0.577	0.564
46+	-0.335	0.124	-2.696	0.007
Race				
Black				
Native American	0.649	0.29	2.24	0.025
Other/Mixed race/Refused	0.397	0.307	1.292	0.196
White	0.388	0.243	1.598	0.11
Male	-0.251	0.085	-2.971	0.003
Hispanic	-0.198	0.228	-0.869	0.385
Homeless in past 6 months	0.255	0.085	3.004	0.003
Injected drugs daily in past 30 days	0.742	0.086	8.607	<.001
Past 30-day drug use				
Methamphetamine	-0.011	0.114	-0.098	0.922
Cocaine	0.071	0.094	0.758	0.448
Fentanyl	0.57	0.099	5.742	<.001
Any opioids	0.396	0.124	3.196	0.001
SD (site-level intercept)	0.679			

© 2023 Hoover DB et al. *JAMA Network Open*.

eTable 5. Model 5: Currently have naloxone

term	estimate	std.error	statistic	p.value
(Intercept)	-2.248			
Recent Incarceration	0.018	0.088	0.205	0.838
Health insurance	0.131	0.102	1.282	0.2
Age				
18-30				
31 - 45	-0.008	0.096	-0.082	0.934
46+	-0.269	0.129	-2.081	0.037
Race				
Black				
Native American	0.713	0.303	2.349	0.019
Other/Mixed race/Refused	0.104	0.329	0.316	0.752
White	0.26	0.265	0.983	0.325
Male	-0.108	0.085	-1.274	0.203
Hispanic	0.045	0.229	0.195	0.845
Homeless in past 6 months	-0.063	0.086	-0.726	0.468
Injected drugs daily in past 30 days	0.785	0.09	8.707	<.001
Past 30-day drug use				
Methamphetamine	-0.079	0.114	-0.691	0.49
Cocaine	0.084	0.094	0.895	0.371
Fentanyl	0.28	0.098	2.862	0.004
Any opioids	0.752	0.144	5.203	<.001
SD (site-level intercept)	0.649			

© 2023 Hoover DB et al. *JAMA Network Open*.

eTable 6. Model 6: Overdose in past 6 months (N = 2867)

term	estimate	std.error	statistic	p.value
(Intercept)	-3.287			
Recent Incarceration	0.32	0.107	3.003	0.003
Health insurance	-0.167	0.118	-1.416	0.157
Age				
18-30				
31 - 45	-0.089	0.114	-0.775	0.439
46+	-0.299	0.166	-1.806	0.071
Race				
Black				
Native American	-0.354	0.359	-0.986	0.324
Other/Mixed race/Refused	-0.294	0.402	-0.731	0.464
White	-0.099	0.304	-0.324	0.746
Male	-0.089	0.104	-0.852	0.394
Hispanic	-0.206	0.315	-0.655	0.513
Homeless in past 6 months	0.439	0.107	4.111	<.001
Injected drugs daily in past 30 days	0.496	0.113	4.388	<.001
Past 30-day drug use				
Methamphetamine	0.344	0.138	2.494	0.013
Cocaine	0.155	0.11	1.406	0.16
Fentanyl	0.7	0.12	5.848	<.001
Any opioids	0.913	0.218	4.182	<.001
SD (site-level intercept)	0.138			

© 2023 Hoover DB et al. *JAMA Network Open*.
