# Supplementary material for: Recent Incarceration, Substance Use, Overdose, and Service Use Among People Who Use Drugs in Rural Communities
Source: JAMA Netw Open. 2023 Nov 9;6(11):e2342222. doi: 10.1001/jamanetworkopen.2023.42222 (PMC10636631; doi:10.1001/jamanetworkopen.2023.42222)
Supplement: Supplement 2. — Data Sharing Statement [file jamanetwopen-e2342222-s002.pdf]

Data Sharing Statement

Hoover. Recent Incarceration, Substance Use, Overdose, and Service Use Among People Who Use Drugs in Rural Communities. *JAMA Netw Open*. Published November 09, 2023. doi:10.1001/jamanetworkopen.2023.42222

Data
Data available: No
